# HIV-1 Intersection with CD4 T Cell Vesicle Exocytosis: Intercellular Communication Goes Viral

**DOI:** 10.3389/fimmu.2014.00454

**Published:** 2014-09-22

**Authors:** Helena Soares

**Affiliations:** ^1^Instituto Gulbenkian de Ciência, Oeiras, Portugal; ^2^Faculdade de Ciências Médicas, CEDOC, Universidade Nova de Lisboa, Lisboa, Portugal

**Keywords:** intercellular communication, immune cell junctions, immunological synapses, extracellular vesicles, HIV-1, virological synapse

## Abstract

In cells of the immune system, the secretion of extracellular vesicles is modulated through cellular activation. In particular, T cell activation is achieved through cell–cell contacts with antigen presenting cells and the consequent formation of a specialized signaling junction called the immunological synapse. Recent works on CD4 T cells have elucidated that cognate antigen recognition by the T cell receptor (TCR) engages two distinct exocytic events. The first involves the exocytic targeting of signaling molecules at the synaptic membrane and drives the functional architecture of the immunological synapse. The second enlists the extracellular secretion of the TCR itself, once the functional architecture of the immunological synapse is accomplished. HIV-1, a human lymphotropic virus, has evolved sophisticated mechanisms to co-opt CD4 T cell physiology. Notably, it has become apparent that HIV-1 intersects the regulated secretory system of CD4 T cells in order to bud from the plasma membrane of the infected cell and to promote bystander cell death. Here, I review the relevance of CD4 vesicle exocytosis to immune regulation and to HIV-1 pathogenesis and discuss their potential therapeutic applications.

## Introduction

The mounting of an immune response relies on the efficiency of intercellular communication. Contact-dependent and -independent intercellular communications are transversal to all cell types. T cells and antigen-presenting cells (APCs) have evolved a refined mechanism for molecular information exchange, which combines both contact-dependent and -independent intercellular communication. The immunological synapse consists of a tightly structured signaling platform induced upon T cell receptor (TCR) recognition of the cognate antigen–MHC complexes presented on the surface of the APC. It functions both as a transducer, converting antigen detection in T cell activation, and as a site of exocytic targeting and secretion of vesicles (1–7). Recent works have begun to underpin the molecular processes that regulate the exocytic targeting and release of different vesicular compartments at the synaptic membrane, as well as their contribution to the synaptic nanostructure and to the coordination of the immune response (1–3). Taken together, the immunological synapse is emerging as a hotspot for vesicle-mediated processes, encompassing exocytic targeting of vesicles containing signaling molecules (1, 2), the polarized secretion of cytokines (3), the release of extracellular vesicles carrying membrane-associated molecules [TCR (4, 5), Fas ligand (FasL) (8), and cytotoxic T lymphocyte antigen-4 (CTLA-4) (9)], and the release of extracellular vesicles containing genetical material in the form of microRNA (siRNA) (6, 7). The integration of the immune response relies on the specificity of the intercellular exchange of a variety of vesicles, transporting transmembrane proteins, genetic information, signaling molecules, and soluble mediators. In particular, in T cells, the release of exocytic vesicles into the synapse is subordinated to TCR engagement, thus adding another dimension to the tight spatiotemporal control of an effective immune response. How exactly the synaptic targeting of a variety of vesicles contributes to the breadth of the adaptive immune response is still an open question.

Co-evolution between viruses and their hosts promotes a highly complex immune system and sophisticated viral mechanism to antagonize immunity. With only 9 genes encoding for 15 proteins, HIV-1 must rely on the hijacking of host–cell proteins to promote its cell-to-cell spread. There is ample evidence that, in T cells, HIV-1 co-opts the polarized exocytic pathway of vesicles in order to increase viral transmission at cellular junctions (5, 10–12), while concomitantly being shielded from immune detection (13). Moreover, HIV-1 promotes the transfer of its entry receptors to cell types that would not otherwise be infected, potentially increasing its dissemination (14). HIV-1 also increases the secretion of immunomodulatory and Nef-containing vesicles (15–18). However, and in a classic example of host–pathogen co-evolution, infected cell vesicle secretion can also be used to deter HIV-1 replication (18).

Recent works have elucidated that the specificity of T cell–APC communication is achieved through the compounded action of the exocytic targeting of signaling molecules and cytokines and through the extracellular vesicle exchange at the immunological synapse. In addition, there is increasing evidence that HIV-1 co-opts CD4 T cells-regulated secretion of extracellular vesicles in order to modulate the host immune response and to increase its propagation. In this review, I will go over the emerging concept of vesicles as important vehicles of communication at cell–cell junctions and their relevance in the modulation of the immune response. I will also address the role of extracellular vesicles in the evolutionary cross-talk between HIV-1 propagation and host defense mechanisms.

## Regulated Vesicle Targeting of Cytokines and Signaling Molecules at the Immunological Synapse

The hallmark of the adaptive system is diversity, the capacity to recognize and to respond to an infinite array of specificities, in an appropriate manner. Consequently, the adaptive immunity has evolved strategies to exert their effector function while minimizing detrimental bystander effects. Earlier work in CD8 T cells had shown that cytotoxic granules are delivered to target cells at the center of the immunological synapse (19). This delivery of lytic granules into the synaptic cleft ensures the specific killing of the target cell without affecting the viability of the bystander cells. Lytic granule fusion at the immunological synapse has been well defined and involves a number of vesicle docking and fusion regulators similar to those found in neural synapses, including synaptotagmin-like calcium sensors (20). CD4 T cells help B cells to make antibodies and orchestrate immune responses through the secretion of panoply of chemokines and cytokines. To an extent, the homeostasis of the immune system relies on the capacity of CD4 T cells to convey a given cytokine to the adequate target cell. The concept that cytokines could be secreted by the CD4 T cell in a regulated and unidirectional manner emerged 20 years ago with the observation that vesicles containing cytokines polarized toward the APC (21). More recently, it was demonstrated that this was the case for cytokines designed to have a local effect, such as IL-2, IL-10, and IFN-γ (3, 22). However, cytokines with inflammatory and chemotactic functions (TNF) do not polarize toward the stimulatory interface and undergo multidirectional secretion (3). Overall, the polarized secretion of cytokines at the synaptic cleft constitutes an exquisite way to ascribe target specificity to soluble proteins.

In addition to the above-described role in T cell effector function, vesicular traffic is also crucial to the formation and regulation of the immunological synapse. A well-characterized signal for regulated vesicle secretion by T cells is TCR engagement by peptide–MHC (pMHC) on the surface of the APC. TCR-mediated calcium flux induces reorientation of the Golgi and secretory vesicles to the T cell–APC junction, enabling the polarized secretion of vesicular cargo, including surface receptors and signaling molecules, toward the engaged target cell. The TCR clustering itself requires the polarized traffic and fusion of TCR vesicles at the synaptic membrane. The fusion of TCR vesicles is mediated by the vSNAREs, VAMP2/cellubrevin, alongside with the synaptic clustering of the plasma membrane tSNAREs, SNAP23, and syntaxin4 (23). Seemingly, reorientation of recycling endosomes toward the T cell–APC contact site synergizes with SNAREs synaptic clustering to ensure TCR delivery at the immunological synapse (2).

It has become increasingly clear that vesicle traffic achieves a variety of effects on signal transduction and, conversely, that receptor signaling regulates the exocytic machinery (24). In the case of the TCR pathway, this signaling/vesicle traffic cross talk includes not only the TCR but also other components of the TCR signaling machinery that traffic in vesicular compartments, which rapidly polarize to the synapse following antigenic stimulation. The protein tyrosine kinase Lck, the adapter LAT, and the TCR subunit TCRζ traffic to the immunological synapse in distinct exocytic vesicles (24). TCR-regulated signal induces LAT vesicles fusion at the synaptic membrane, where LAT phosphorylation ensues (24). It can be argued that TCR engagement drives the reorganization of tSNARE microclusters (23, 25), which enables the exocytic targeting of the TCR and its downstream signaling molecules Lck, LAT, and TCRζ at the synaptic membrane (24). TCR signaling is not merely a function of which signaling molecules are present at the synaptic membrane but also a function of their subcellular origin and traffic regulation (24). The compartmentalization of signaling molecules into distinctly regulated vesicle compartments and the establishment of a defined spatial organization for vesicle fusion at the synaptic membrane is expected to contribute to determine the activation breadth in response to different stimulatory conditions.

In conclusion, underpinning both T cell activation and effector function resides a series of regulated vesicle fusion events accountable for the spatiotemporal organization of the TCR signaling and polarized cytokine secretion, respectively.

## CD4 T Cell Secretion of Extracellular Vesicles at the Immunological Synapse

Extracellular vesicles are known to be potent modulators of the immune system. Their immunomodulatory function ranges from tolerance to the fetus during pregnancy (26), to mediating immune escape, and to the priming of an effective immune response (27). While the release of extracellular vesicles by dendritic cells has been extensively documented (27), the secretion of extracellular vesicles by CD4 T cells has lagged behind.

CD4 T cells release of extracellular vesicles has been linked to TCR activation (28). Activated CD4 T cells were first reported to secrete extracellular vesicles containing FasL and APO2 ligand, in a putative mechanism to maintain T cell homeostasis and immune tolerance through the apoptosis of targeted cells (29). Fitting with its functions as a device for intercellular information transfer (30), the secretion of TCR containing vesicles has been found to require the establishment of an immunological synapse (4, 5). The requirement of immunological synapse for the release of TCR-containing vesicles confers spatiotemporal specificity and directionality to the process, making them powerful vehicles to specifically deliver signals to cognate APCs. The physiological function of TCR secretion into extracellular vesicles remains an open question. At least two, not mutually exclusive, possibilities can be envisioned to: (i) downregulate the T cell activation; (ii) allow for a distinct pattern of integration of activation signals by the cognate APC.

Mittelbrunn et al. proposed that T cells can extend the limits of the transcriptome of their cognate APC by transferring vesicles containing genetic material (miRNA) (6, 7). This transfer was found to be unidirectional, from the engaged T cell to the cognate APC, and dependent on immunological synapse formation (6, 7). A very recent work has shown that extracellular vesicles containing miRNA are also transferred between T cells (31). Regulatory CD4 T cells transfer miRNA containing extracellular vesicles to T helper type 1 (Th1) cells, both *in vivo* and *in vitro* (31). The transferred miRNA suppressed both Th1 cell proliferation and IFN-γ secretion (31). It is still unclear if, analogous to T cell–APC vesicle transfer (4, 5, 7), the T cell-to-T cell exchange also requires a form of organized cell contact.

In eukaryotic organisms, extracellular vesicles are increasingly recognized as important mediators of cell-to-cell communication. However, exactly how the extracellular vesicles deliver their content to their target cells is still unclear. Extracellular vesicles emanating from immune cells face a particular challenge in order to achieve target specificity in the cell-dense environment of secondary lymphoid organs. It is possible that CD4 T cells, as well as other immune cells, have evolved to precise their extracellular vesicles delivery route via immunological synapse. The immunological synapse could act as a highly efficient platform that directs antigen-specific delivery of extracellular vesicles to the specific target APC, without affecting surrounding bystander cells.

## HIV-1 and CD4 T Cell Vesicle Exocytosis

HIV-1 infects CD4 T cells, eludes the CD4 T cell immune response, and finally, elicits an inflammatory immune response that ultimately leads to CD4 T cell exhaustion and unresponsiveness. The infectivity of cell-associated HIV-1 is much higher than that of cell-free virus and impervious to neutralizing antibodies (32, 33). As such, HIV-1 has evolved to hijack the mechanisms of intercellular communication to favor its dissemination. In common with other enveloped viruses, HIV-1 hijacks the cellular vesiculation machinery for the budding of newly formed infective virions (34, 35). It has become apparent that not only HIV-1 uses the vesiculation pathway for its own assembly and release but also exploits the transfer of extracellular vesicles at the immune junctions. A cell–cell junction particularly relevant for HIV-1 spread is the virological synapse. Akin to the immunological synapse, the virological synapse is a molecularly organized cell–cell junction, formed between an infected and an uninfected CD4 T cells, that allows for efficient HIV-1 transmission (36, 37). The formation of the virological synapse promotes the reorientation of the infected cell exocytic machinery toward the cell junction and the consequent of egress of HIV-1 at the sites of cell–cell contact (10, 11, 38). Moreover, vesicles that are targeted at the immunological synapse also polarize to the virological synapse (12). Seemingly, HIV-1 hijacks the regulated vesicle targeting of cytokines and signaling molecules from the immunological synapse to the virological synapse, in order to increase its dissemination.

In CD4 T cell physiology, the bonafide cell–cell contact that elicits vesicle exocytosis is the immunological synapse. Engagement of the TCR has been shown to trigger the exocytosis of extracellular vesicles enriched for the TCR at the synaptic cleft (4, 5). A recent paper has shown that HIV-1 gag outcompetes the TCR for its sorting in extracellular vesicles. In gag expressing CD4 T cells, TCR engagement conduces to the polarized budding of virus like particles at the immunological synapse (5). In this context, antigen recognition by the HIV-1 infected T cell could play an important role in the generation of viral depots in cognate macrophages and dendritic cells. By connecting its budding to TCR engagement, HIV-1 restricts the release of infective virions to the window of time where a cell junction is formed. This tight spatiotemporal control of HIV budding upon cognate immune interactions could potentially favor the more efficient cell-associated virus transmission, while limiting the release of the less infective cell-free virions. It remains to be addressed if immunological synapse formation increases the susceptibility of the APCs to HIV-1 infection.

The role of extracellular vesicles in HIV-1 infection surpasses HIV-1 budding; extracellular vesicles contribute to an upsurge in both HIV-1 infectivity and in immune response (14, 18). For one, HIV-1 broadens its cellular tropism by promoting the release of vesicles carrying its entry co-receptor-CCR5 to originally non-permissive cells (14). The secretion of extracellular vesicles by HIV-1 infected cells appears to be a concerted strategy to involve bystander cells in the course of HIV-1 infection. The HIV-1 protein Nef causes massive secretion of extracellular vesicles (15) including Nef containing ones (16, 39). Nef containing extracellular vesicles might contribute to HIV-1 immunodeficiency by inducing the apoptosis of bystander T cell (16). Nonetheless, the secretion of extracellular vesicles can also promote innate immune responses to HIV-1 infection. APOBEC3G, a HIV-1 postentry restriction factor (40), is able to inhibit HIV-1 replication upon transfer to recipient cells via extracellular vesicles (18). HIV-1 not only co-opts extracellular vesicles for its assembly and release but is also capable of exploiting the highly complex role of extracellular vesicles in immune cell communication.

## Discussion and Perspectives

The host–pathogen relationship is a dynamic process in which the pathogen attempts to minimize its detection, whereas the host attempts to prevent and eradicate infection with minimal damage to self. The host responds to infection by mounting an immune response whose efficiency relies on the intercellular communication between the immune cells. Communication between immune cells involves the formation of the immunological synapse that stabilizes TCR engagement for the time required for gene transcription and for the secretion of soluble mediators and the transfer of extracellular vesicles containing immune receptors and gene editing molecules (2, 6, 24). At the immunological synapse, membrane delivery and removal occur in coordinated fashion to ensure a balanced cycling of membranes (41). Hence, the delivery of vesicles carrying signaling molecules and the release of TCR exocytic vesicles co-occur at the synaptic membrane (5, 24). It is possible that the functional consequences of these membrane transfers at the immunological synapse include not only the diversification and amplification of the T cell activation but also the immune modulation of the cognate APCs (5, 24). A distinct effect of synapse formation is the polarized secretion of cytokines and mRNA containing extracellular vesicles (3, 7). In the crowded environment of the secondary lymphoid organs, directionality might be critical for conveying T cell effector activities to the target cell of the correct antigen specificity by avoiding at once, effects on bystander cells and the dissipation of these mediators in the interstitial space.

In the secondary lymphoid organs, prolonged contacts between T cells and APCs might promote other types of cell–cell synapses. In fact, T cell–T cell contacts have been described to play an important role in the priming of a protective immune response (42). Accordingly, full suppression activity by miRNA containing vesicles required the presence of Tregs (31). Indicating that Treg suppression via the transfer of miRNA containing extracellular vesicles could rely on T cell–T cell contacts in order to silence the genetic program of the target cell.

The exact mechanism of extracellular vesicle transfer at the immunological synapse still remains to be determined. It is possible that, akin to cytotoxic CD8 T cells, vesicle transfer occurs through the immunological synapse itself (43). Alternatively, extracellular vesicles could be released to the extracellular space in the vicinity of the immunological synapse, creating a trail of immunoreceptor-enriched vesicles with increased probability to be transferred to the engaged APC (5). This latter scenario could help explain the migratory patterns of APCs and naïve T cells alike in the draining lymphoid organs (44). Synaptic-dependent release of immunoreceptors containing vesicles could guide the encounter APCs and T cells of a rare specific antigenic specificity, thus promoting an exceedingly rare event if left to occur randomly.

Co-evolution between viruses and their hosts promoted a highly complex host immune system and sophisticated viral mechanisms to antagonize immunity. HIV-1 infection leads to a strong alteration of infected T cell physiology, promoting efficient viral replication, T cell depletion, and immunodeficiency. Since T cell-derived extracellular vesicles coordinate the immune response against infection, HIV-1 has, during its evolution, forcibly probed for key vesiculation checkpoints to better suit its replication and cell-to-cell transmission. By co-opting CD4 T cell extracellular vesicles, HIV-1 is able to or control the status of immune activation, even in the absence of viral replication. HIV-1 co-opts the function of extracellular vesicles in immune modulation (16), in intracellular communication (14), and for cell to cell transfer (5, 34, 35). Thus, HIV-1 hijacks the CD4 T cell vesiculation machinery not only for its own assembly and budding but also to modulate the sophisticated communication system executed by extracellular vesicles. The two most obvious outcomes are an increase in the efficiency of HIV-1 cell-to-cell transmission, by limiting HIV-1 budding to productive cell-to-cell contacts with permissive target cells (5, 34, 35), and recruiting bystander cells to the compounding HIV-1-driven immune dysregulation (15, 18). More research is needed to address if extracellular vesicles originated by distinct CD4 T cells subsets convey differential immune functions, and how their cross talk contributes for the regulation of the immune response and for the outcome of HIV-1 infection.

In addition to the increasing interest to screen for extracellular vesicles as biomarkers of disease, investigating HIV-1 intersection with the CD4 T cell vesiculation machinery will increase our understanding of these two processes and might contribute to identify novel anti-retroviral targets.

## Conflict of Interest Statement

The author declares that the research was conducted in the absence of any commercial or financial relationships that could be construed as a potential conflict of interest.
